# Qualitative and Quantitative Analysis of the Major Bioactive Components of *Juniperus chinensis* L. Using LC-QTOF-MS and LC-MSMS and Investigation of Antibacterial Activity against Pathogenic Bacteria

**DOI:** 10.3390/molecules28093937

**Published:** 2023-05-07

**Authors:** Da Jung Lim, Jeong-Sup Song, Byoung-Hee Lee, Youn Kyoung Son, Yangseon Kim

**Affiliations:** 1 Department of Research and Development, Center for Industrialization of Agricultural and Livestock Microorganisms, Jeongeup-si 56212, Republic of Korea; limdajung@cialm.or.kr (D.J.L.); jungsup717@cialm.or.kr (J.-S.S.); 2 Biological Resources Assessment Division, National Institute of Biological Resources, Incheon 22689, Republic of Korea; dpt510@korea.kr

**Keywords:** *Juniperus chinensis*, flavonoids, amentoflavone, quercetin-3-*O*-α-l-rhamnoside, antibacterial activity

## Abstract

Plants in the genus *Juniperus* have been reported to produce a variety of chemical components, such as coumarins, flavonoids, lignans, sterols, and terpenoids. Here, ultra-high-performance liquid chromatography coupled with quadrupole time-of-flight mass spectrometry (UPLC-QTOF-MS) and ultra-high-performance liquid chromatography-tandem mass spectrometry (UPLC-MS/MS) were applied to qualitatively and quantitatively analyze the major bioactive components in an ethanolic crude extract from the leaves of *Juniperus chinensis* L., which grows naturally in Korea. In addition, the antibacterial activity of the crude extract against pathogenic bacteria was investigated. Using LC-QTOF-MS analysis, we identified ten compounds, of which six were confirmed to be flavonoid and lignan-based components as the major bioactive components, i.e., isoquercetin, quercetin-3-*O*-α-l-rhamnoside, hinokiflavone, amentoflavone, podocarpusflavone A, and matairesinoside. Among them, a quantitative analysis performed using LC-MS/MS revealed that the levels of quercetin-3-*O*-α-l-rhamnoside and amentoflavone in the crude extract were 203.78 and 69.84 mg/g, respectively. Furthermore, the crude extract exhibited potential antibacterial activity against 10 pathogenic bacteria, with the highest antibacterial activity detected against *Bordetella pertussis*. Thus, further studies of the leaf extract of *J. chinensis* L. must be carried out to correlate the compounds present in the extract with the antibacterial activity and elucidate the mechanisms of action of this extract against bacteria.

## 1. Introduction

*Juniperus chinensis* L. is known as Chinese juniper and is commonly found in many regions in Asia, including China, Taiwan, Myanmar, Japan, Malaysia, and Korea [1]. The genus *Juniperus* is considered as an important source of medicinal plants that are rarely used in traditional medicine. Various *Juniperus* species have been reported to produce a variety of chemical components, such as coumarins, flavonoids, lignans, sterols, and terpenoids [2], with a wide range of medicinal properties, such as antimicrobial, antioxidant, antitumor, and anticancer effects [3,4,5,6].

Flavonoids or bioflavonoids are a class of polyphenolic secondary metabolites that are found in plants and fulfill many functions [7]. The basic structure of these compounds consists of a general structure of 15 carbons with two phenyl rings and a heterocyclic ring, termed diphenylpropane (C6-C3-C6) skeleton [8]. Flavonoids are especially well known as having antibacterial properties against many pathogenic microorganisms. Recently, several investigations were performed regarding the antimicrobial activities, also summarizing the probable relationships between the chemical structures and antimicrobial activities [8]. These compounds exert their antibacterial effects via a mechanism mainly targeting the cell membrane, which likely involves the inhibition of attachment and biofilm formation, the inhibition of phospholipid bilayer formation, the inhibition of the respiratory chain, ATP-synthesis-mediated damage to the energy metabolism, etc. [7,9,10]. Lignans are a class of polyphenolic secondary metabolites that are found in plants and are precursors to phytoestrogens. These compounds have a general basic structure of nine carbons consisting of two phenylpropane (C6-C3) skeletons [11] and have been reported to have anticancer, antioxidant, antimicrobial, anti-inflammatory, and immunosuppressive activities [12,13,14].

In the present study, we qualitatively analyzed the bioactive components of a crude extract of *J. chinensis* L. using ultra-high-performance liquid chromatography coupled with quadrupole time-of-flight mass spectrometry (UPLC-QTOF-MS). Among them, quantitative analysis was performed for those with a mass spectrum, fragmentation pattern, and retention time which matched the reference compounds based on liquid chromatography-tandem mass spectrometry (LC-MS/MS). Furthermore, the antibacterial activity of the crude extract against 10 pathogenic bacteria was evaluated.

## 2. Results and Discussion

### 2.1. Chemical Profiling of Juniperus chinensis L.

**Figure 1:**
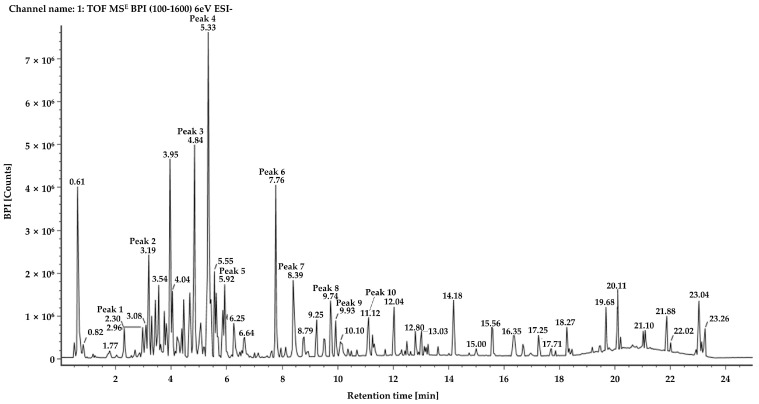
Base peak ion chromatogram of the *J. chinensis* L. extract obtained by UPLC-QTOF-MS analysis.

**Figure 2:**
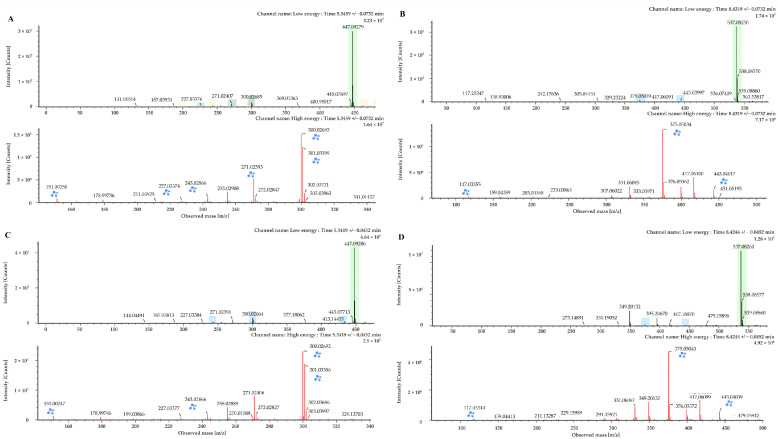
Comparison of reference materials quercetin-3-*O*-α-l-rhamnoside (**A**) and amentoflavone (**B**) to identify the components of peak 4 (**C**) and peak 7 (**D**) using mass spectra obtained by UPLC-QTOF-MS.

**Figure 3:**
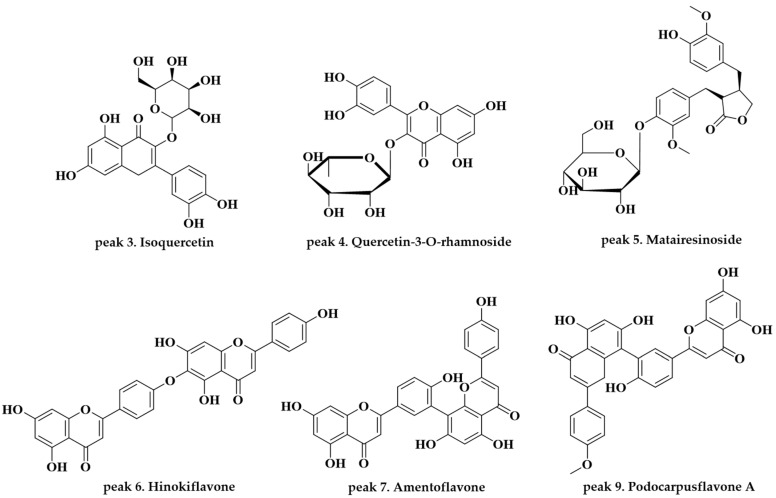
Chemical structures of the components identified in the ethanolic extract of *J. chinensis* L. based on the UPLC-QTOF-MS analysis.

An ethanolic crude extract of the leaves of *J. chinensis* L. was analyzed and characterized using UPLC-QTOF-MS in electrospray ionization (ESI) negative ion mode, because more information and a higher fragmentation could be obtained in this ion mode compared with positive ion mode. We investigated various physiologically active component peaks in the base peak ion chromatogram, as shown in Figure 1. Ten components were tentatively identified after a library search using Waters’ UNIFI software (version 1.9, Milford, MA, USA) and the ChemSpider online database of isolated components under chromatographic conditions. All components exhibited a mass error below 5 mg/kg (Table 1, Supplementary Figure S1). Among them, the components corresponding to peaks 4 and 7 were identified as quercetin-3-*O*-α-l-rhamnoside and amentoflavone, respectively, based on reference compounds with mass spectra with identical patterns (Figure 2). Peak 1 (observed RT: 2.31 min; formula: C_21_H_24_O_11_), Peak 2 (observed RT: 3.19 min, formula: C_15_H_14_O_6_), Peak 8 (observed RT: 9.76 min; formula: C_20_H_16_O_7_), and Peak 10 (observed RT: 11.12 min; formula: C_17_H_26_O_4_) detected in the crude extract were identified via a tentative investigation based solely on formulas. In contrast, other components were unambiguously identified by comparison with reference substances. The structures of those components are shown in Figure 3.

Peak 3 (observed RT: 4.84 min; formula: C_21_H_20_O_12_) was identified as isoquercetin, as reported in [15,16]. The precursor ion of this component was detected at *m*/*z* 463.0878 [M − H]^−^ ion, with a fragment ion at *m*/*z* 300.0267 [463–C_6_H_12_O_5_]^−^ with the main peak. Isoquercetin has been reported to inhibit the oxidative stress effect of multiple carcinogens [21,22]. Peak 4 (observed RT: 5.34 min; formula: C_21_H_20_O_11_) was identified as quercetin-3-*O*-α-l-rhamnoside, as reported in [16,17,18]. The precursor ion of this component was detected at *m*/*z* 447.0929 [M − H]^−^ with fragment ions at *m*/*z* 300.0266 [447–C_6_H_12_O_4_]^−^ and 243.0299 [447–C_8_H_13_O_6_]^−^ and the main peak at *m*/*z* 300.0266. Quercetin-3-*O*-α-l-rhamnoside has been reported to have cytotoxic effects and antibacterial and antioxidant activities [23,24,25]. Peak 5 (observed RT: 5.94 min; formula: C_26_H_32_O_11_) was identified as matairesinoside, as reported in [19]. The precursor ion of this component was detected at *m*/*z* 519.1870 [M − H]^−^ with fragment ions at *m*/*z* 357.1340 [519–C_6_H_11_O_5_]^−^ and 343.1104 [519–C_7_H_14_O_5_]^−^ and the main peak at *m*/*z* 357.1340. Matairesinoside has been reported to have cytotoxic effects and antibacterial activity [26]. Peak 6 (observed RT: 7.78 min; formula: C_30_H_18_O_10_) was identified as hinokiflavone, as reported in [20]. The precursor ion of this component was detected at *m*/*z* 537.0826 [M − H]^−^ with fragment ions at *m*/*z* 443.0489 [537–C_6_H_7_O]^−^, 375.0510 [537–C_9_H_7_O_3_]^−^, and 117.0346 [537–C_22_H_13_O_9_]^−^ and the main peak at *m*/*z* 375.0510. Hinokiflavone has been reported to have cytotoxic effects and anti-inflammatory and antioxidant activity [27,28]. Peak 7 (observed RT: 8.42 min; formula: C_30_H_18_O_10_) was identified as amentoflavone, as reported in [16,18,20]. The precursor ion of this component was detected at *m*/*z* 537.0826 [M − H]^−^ with fragment ions at *m*/*z* 443.0409 [537–C_6_H_7_O]^−^ and 375.0510 [537–C_9_H_7_O_3_]^−^ and the main peak at *m*/*z* 375.0510. Amentoflavone has been reported to have various bioactivities, including antioxidant, anti-inflammatory, anti-senescence, antitumor, anti-virus, and anti-fungal effects [29,30,31,32]. Peak 9 (observed RT: 9.97 min; formula: C_31_H_20_O_10_) was identified as podocarpusflavone A, as reported in [20]. The precursor ion of this component was detected at *m*/*z* 551.0984 [M − H]^−^ with fragment ions at *m*/*z* 519.0713 [551–CH_5_O]^−^ and 375.0506 [551–C_21_H_11_O_7_]^−^ and the main peak at *m*/*z* 375.0506. Podocarpusflavone A has been reported to have cytotoxic effects and antimicrobial activity [33,34]. Of the 10 compounds identified in this study, six were confirmed to be flavonoid and lignan-based components which have diverse physiological activities according to the literature.

### 2.2. Quantitative Analysis of the Reference Compounds

The two reference compounds, quercetin-3-*O*-α-l-rhamnoside and amentoflavone, were analyzed using UPLC-Xevo TQ-S micro MS/MS in ESI mode. The analytical conditions of the instrument were set so that the retention time of the analyte was the same on the chromatograms of the standard solutions and the sample solutions. The ion ratio was calculated as the peak area ratio between a less intense ion and a more intense ion. The reference ion ratio value was calculated as the average of the ion ratios of the calibration solutions. All samples met the instrument analytical conditions described above (not shown). Moreover, the quercetin-3-*O*-α-l-rhamnoside standard calibration revealed a coefficient of determination (R^2^) of 0.9991 with good linearity, ranging from 0.05 to 1 ng. The amentoflavone standard calibration revealed a coefficient of determination (R^2^) of 0.9955 with good linearity, ranging from 0.25 to 2.5 ng. Overall, these results indicated that the methods were sufficiently validated to identify the two reference compounds in the samples, as a result of the standard curves of quercetin-3-*O*-α-l-rhamnoside and amentoflavone and the measurement of the total compound content (Table 2). The total contents (weight) in the ethanolic extract of quercetin-3-*O*-α-l-rhamnoside and amentoflavone were 203.78 and 69.84 mg/g, respectively.

### 2.3. Antibacterial Analysis

The antibacterial activities of the ethanolic crude extract of the leaves of *J. chinensis* L. were evaluated against pathogenic strains. The ethanolic crude extract of *J. chinensis* L. exhibited antibacterial activity against all strains tested here (Table 3). The inhibition zones were similar to or lower than those of antibiotics. Our results showed activity for both gram-positive and gram-negative pathogens similar to those observed in the literature [2,35,36]. For *E. coli* and *S. aureus*, antibacterial activity was observed among various *Juniperus* leaves and fruits [35,36]. Ennajar M et al. showed intense activity for *K. pneumoniae*, while our results showed only low activity [37]. On the other hand, the crude extract of *J. chinensis* L. exhibited strong antimicrobial activity against *Bordetella pertussis* NCCP13671.

## 3. Materials and Methods

### 3.1. Plant Material

The leaves of *Juniperus chinensis* L. were collected on 6 November 2018 from Imhyeon-ri, Eosangcheon-myeon, Danyang-gun, Chungcheongbuk-do, South Korea. The National Institute of Biological Resources (NIBR) performed the botanical identification of the plant materials. Specimens and materials of *J. chinensis* L. were deposited in the Wildlife Natural Products Bank at the NIBR, with NIBR numbers NIBRVP0000725557 and NIBRGR0000611643, respectively.

### 3.2. Preparation of the Plant Extract

The leaves of *J. chinensis* L. were collected to prepare the ethanolic extract. The leaves were dried in an oven (HB-503SF, HANBAEK) at 40 °C for 48 h and then ground to a fine powder to pass through a 20-mesh sieve. The powder was extracted with 70% ethanol (Sigma-Aldrich, Saint Louis, MO, USA) at four times the volume (1:4, *v*/*v*). The mixture was left at room temperature for 72 h, centrifuged at 4000× *g* for 10 min (VARISPIN 15R, CRYSTE), and filtered using filter paper (Whatman No. 2, 4.25 cm diameter). Moreover, the solvent was concentrated by rotary evaporation (Rotavapor R-100, Buchi, Flawil, Switzerland). The crude extract was stored at −80 °C in a freezer until it was used for analysis of the major bioactive components and antibacterial activity.

### 3.3. Qualitative Analysis Using Ultra-High-Performance Liquid Chromatography-Quadrupole Time-of-Flight Mass Spectrometry

The chemical profiling analysis of the crude extract from *J. chinensis* L. was performed using Ultra-High-Performance Liquid Chromatography-Quadrupole Time-of-Flight Mass Spectrometry (UPLC-QTOF-MS) in negative modes using an ACQUITY^TM^ UPLC-Xevo GS-XS Q-TOF (Waters) instrument equipped with an ACQUITY UPLC^®^ BEH C18 column (100 × 2.1 mm, 1.7 µm; Waters, Milford, MA, USA) at 40 °C. The mobile phase consisted of 0.1% formic acid in water (A) and acetonitrile (B) at a flow rate of 0.4 mL/min, and the injection volume was set at 1 µL. At this time, the injected sample was diluted to 2500 mg/L level with LC grade methanol. The mobile phase was a gradient: 0–1 min, 5% solvent B; 1–20 min, 100% solvent B; 20–20.30 min, 100% solvent B; 22.30–22.40 min, 5% solvent B; and 22.40–25 min, 5% solvent B. Full-scan mass spectra were acquired to detect the mass-to-charge ratio (*m*/*z*) in the range of 50–1500. The detector conditions were optimized as follows: the source temperature was set at 120 °C with a capillary voltage of 2.5 kV. Data acquisition and analysis were controlled using the traditional medicine library in the Waters’ UNIFI software (version 1.9, Milford, MA, USA). First, the identification of the components was performed by checking the allowable chemical formula calculated for the mass error range ± 5 ppm and setting it to fragment tolerance 10 mDa to check the mass pattern. In addition, if the fragment ion pattern did not match, it was finally identified using the Chemspider (http://www.chemspider.com/, accessed on 27 September 2022) online database. 

### 3.4. Quantitative Analysis Using Ultra-Performance Liquid Chromatography-Tandem Mass Spectrometry

A quantitative analysis was performed using an ACQUITYTM UPLC-Xevo TQ-S micro MS/MS instrument (Waters). Among the major chemical compounds identified through LC-TOF-MS, two chemical compounds, peak 4 (quercetin-3-*O*-α-l-rhamnoside) and peak 7 (amentoflavone), which had the same mass spectra, fragmentation patterns, and retention times as the reference compounds, were quantitatively analyzed. Peak 4 had the following analytical conditions: the mobile phase consisted of 0.1% formic acid and 5 mM ammonium acetate in water (A) and methanol (B) at a flow rate of 0.3 mL/min, with the injection volume set at 2 µL. The mobile phase was a gradient: 0–1 min, 30% solvent B; 1–2 min, 70% solvent B; 3.5 min, 70% solvent B; 3.5–4 min, 30% solvent B; and 4–5 min, 30% solvent B using a CORTECSTM UPLC C18+ column (100 × 2.1 mm, 1.6 µm; Waters) at 35 °C. The MS conditions were optimized as follows: capillary voltage (0.8 kV), source temperature (150 °C), desolvation temperature (350 °C), desolvation gas flow (650 L/h), and cone gas flow (50 L/h), and the system was operated in electrospray ionization (ESI) positive ion mode. Peak 7 had the following analytical conditions: the mobile phase consisted of 0.1% formic acid in water (A) and methanol (B) at a flow rate of 0.5 mL/min, with the injection volume set at 5 µL. The mobile phase was a gradient: 0–0.5 min, 30% solvent B; 0.5–1 min, 70% solvent B; 2 min, 70% solvent B; 2–2.5 min, 100% solvent B; 3.5 min, 100% solvent B; 3.5–4 min, 30% solvent B; and 5 min, 30% solvent B using a CAPCELL CORE C18 column (150 × 2.1 mm, 2.7 µm; Osaka Soda, Shiseido) at 35 °C. The MS conditions were optimized as follows: capillary voltage (2.5 kV), source temperature (150 °C), desolvation temperature (300 °C), desolvation gas flow (450 L/h), and cone gas flow (10 L/h), and the system was operated in ESI negative ion mode. The multiple reaction monitoring for the quantitative mass ion analysis is presented in Table 4. Data acquisition and analysis were controlled using Waters’ Mass Lynx (version 4.2, Milford, MA, USA).

### 3.5. Reference Compounds and Preparation of the Standard Solution

Reference compound quercetin-3-*O*-α-l-rhamnoside (98% purity) was purchased from LGC standard, whereas amentoflavone (98% purity) was purchased from Sigma-Aldrich. The reference compounds were prepared by dissolving them in methanol at a final concentration of 1000 mg/L. The standard solutions were diluted to obtain calibration curves with five points in the concentration range of 0.025–0.5 and 0.05–0.5 mg/L, respectively.

### 3.6. Antibacterial Analysis

The antibacterial activity of the crude ethanol extract from *J. chinensis* L. was evaluated against various pathogenic bacterial strains using a modified disk diffusion method [38]. This study used 10 pathogenic bacteria strains deposited in NCCP or ATCC to conduct the antibacterial analysis: *Escherichia coli* KCTC 2617, *Salmonella enterica* serovar Enteritidis NCCP 14546, *Streptococcus mutans* KCTC 3065, *Staphylococcus aureus* NCCP 14560, *Acinetobacter baylyi* ATCC 33305, *Klebsiella pneumoniae* NCCP 16052, *Bordetella pertussis* NCCP13671, *Moraxella catarrhalis* ATCC 43628, *Staphylococcus pyrogenes* NCCP14783, and *Streptococcus pneumoniae* NCCP 14774. Each bacterial pathogenic strain was grown on suitable media at 30 °C–37 °C for 20 h. *E. coli*, *S. aureus*, and *K. pneumoniae* were grown on nutrient agar; *S. enteritidis* on tryptic soy agar; *S. mutans*, *A. baylyi*, *M. catarrhalis*, and *S. pyrogenes* on brain heart infusion agar; *B. pertussis* on Bordet–Gengou agar; and *S. pneumoniae* on sheep blood agar. To carry out the disc diffusion test as an antimicrobial disc susceptibility test according to the guidelines of the Clinical & Laboratory Standard Institute, each agar plate medium was inoculated with pathogenic bacteria at a concentration of 1–2 × 10^8^ CFU/mL. Sterilized paper discs (8 mm) were placed on the agar, and 10 µL of the crude plant extract was used to impregnate the discs. The extracts were dissolved in DMSO and ethanol (1:1, *v*/*v*) at a concentration of 20 mg/mL. The culture was incubated at 30 °C–37 °C for 24 h, and the diameters of the inhibition zones that formed around each disk were measured in millimeters. The positive control consisted of the appropriate antibiotic for each pathogen, i.e., ampicillin (2.5 mg/mL) for *E. coli*, *S. enteritidis*, *S. mutans*, *K. pneumoniae*, *B. pertussis*, and *S. pneumoniae;* gentamycin (2.5 mg/mL) for *A. baylyi;* penicillin (2.5 mg/mL) for *M. catarrhalis* and *S. pyrogenes*; and kanamycin (50 mg/mL) for *S. aureus*. In contrast, the negative control consisted of DMSO and ethanol (1:1, *v*/*v*). Each experiment was performed in triplicate.

## 4. Conclusions

In the present study, various chemical components were identified in an ethanolic crude extract of the leaves of *J. chinensis* L., which grows naturally in Korea. We identified 10 flavonoid and lignan-based molecules via LC-QTOF-MS analysis as the major bioactive components, including isoquercetin, quercetin-3-*O*-α-l-rhamnoside, hinokiflavone, amentoflavone, podocarpusflavone A, and matairesinoside. Among them, quercetin-3-*O*-α-l-rhamnoside and amentoflavone were quantitatively analyzed using LC-MS/MS; they existed in the crude extract at 203.78 and 69.84 mg/g, respectively. Moreover, the ethanolic crude extracts showed overall inhibitory activities against all Gram-positive and Gram-negative pathogenic bacteria tested, with the highest activity observed against *Bordetella pertussis*. Based on these results, we concluded that the *J. chinensis* L. extract showed antibacterial activity against various pathogens. However, we did not directly check if the 10 components identified here had antibacterial activity. Therefore, further studies are required to assess the antibacterial activity of the compounds isolated from *J. chinensis* L.

## Figures and Tables

**Table 1:** Tentative identification of the chemical components of the ethanolic extract of *J. chinensis* L. obtained from the UPLC-QTOF-MS analysis.

Peak	Tentative Chemical Component	Formula	Observed RT (min)	Neutral Mass (Da)	Observed [M − H]^−^ (*m*/*z*)	Mass Error (ppm)	Fragmentation Peaks *m*/*z* (% Base Peak)	References
1	Unknown 1	C_21_H_24_O_11_	2.31	452.1319	451.1242	−0.9	289.0709(100); 137.0232(100)	-
2	Unknown 2	C_15_H_14_O_6_	3.19	290.0790	289.0712	−2	245.0810(78); 137.0232(100)	-
3	Isoquercetin	C_21_H_20_O_12_	4.84	464.0955	463.0878	−0.9	300.0267(100)	[15,16]
4	Quercetin-3-*O*-α-l-rhamnoside	C_21_H_20_O_11_	5.34	448.1006	447.0929	−1	300.0266(100); 243.0299(7)	Standard [16,17,18]
5	Matairesinoside	C_26_H_32_O_11_	5.94	520.1945	519.1870	−0.3	357.1340(100); 342.1104(7)	[19]
6	Hinokiflavone	C_30_H_18_O_10_	7.78	538.0900	537.0826	−0.2	443.0489(13); 375.0510(100); 117.0346(2)	[20]
7	Amentoflavone	C_30_H_18_O_10_	8.42	538.0900	537.0826	−0.2	443.0409(14); 375.0510(100)	Standard [16,18,20]
8	Unknown 3	C_20_H_16_O_7_	9.76	368.0896	367.0819	−1.3	323.0925(85); 294.0884(100); 159.0452(97)	-
9	Podocarpusflavone A	C_31_H_20_O_10_	9.97	552.1056	551.0984	0	519.0713(2); 375.0506(100)	[20]
10	Unknown 4	C_17_H_26_O_4_	11.12	294.1831	293.1752	−2.1	249.1853(69); 193.1598(100)	-

**Table 2:** Results of the investigation of linear relationships and quantification based on UPLC-MS/MS analysis.

Parameter	Quercetin-3-*O*-α-l-rhamnoside	Amentoflavone
Coefficients of regression equation	y = 34,990x − 205.23	y = 3991.5x + 404.2
Coefficients of determination (R^2^)	0.9991	0.9955
Linear range (ng)	0.05–1.0	0.25–2.5
Concentration (mg/g of dry ethanolic extract weight)	203.78 ± 5.42	69.84 ± 1.94

**Table 3:** Antibacterial activity of the ethanolic crude extract of *J. chinensis* L. against pathogenic bacterial strains.

Test Pathogenic Strain	Antibacterial Activity *		
	Ethanolic Crude Extract of *J. chinensis*	Positive Control	
*Escherichia coli* KCTC 2617	++	++	ampicillin
*Salmonella enterica* serovar Enteritidis NCCP 14546	+	+++	ampicillin
*Streptococcus mutans* KCTC 3065	++	+++	ampicillin
*Staphylococcus aureus* NCCP 14560	++	+++	kanamycin
*Acinetobacter baylyi* ATCC 33305	+	++	gentamycin
*Klebsiella pneumoniae* NCCP 16052	+	++	ampicillin
*Bordetella pertussis* NCCP13671	+++	+++	ampicillin
*Moraxella catarrhalis* ATCC 43628	+	+++	penicillin
*Staphylococcus pyrogenes* NCCP14783	++	++	penicillin
*Streptococcus pneumoniae* NCCP 14774	++	++	ampicillin

* The inhibition zone (mm) around the paper disc containing the microbial cell-free supernatant was classified as follows: +++, >13 mm; ++, 10–12 mm; +, less than 9 mm. All microbial pathogens showed no inhibition against the negative control (DMSO:EtOH = 1:1, *v*/*v*).

**Table 4:** UPLC-MS/MS instrument conditions for the analysis of compounds in multiple reaction mode.

Component	Ion Mode	Precursor Ion *m*/*z*, (Cone Voltage, V)	Product Ions *m*/*z*, (Collision Energy, V)	
			Quantitation	Reference
Quercetin-3-*O*-α-l-rhamnoside	positive	449.18 (20)	303.11 (9)	84.94 (9)
Amentoflavone	negative	537.48 (9)	375.31 (33)	443.34 (33)

## Data Availability

Not applicable.

## Supplementary Materials

The following supporting information can be downloaded at: https://www.mdpi.com/article/10.3390/molecules28093937/s1, Figure S1: Fragment ion pattern isoquercetin (A), quercetin-3-*O*-α-l-rhamnoside (B), matairesinoside (C), hinokiflavone (D), amentoflavone (E), and podocarpusflavone A (F) using mass spectra obtained by UPLC-QTOF-MS.
