# Supplementary material for: Qualitative and Quantitative Analysis of the Major Bioactive Components of Juniperus chinensis L. Using LC-QTOF-MS and LC-MSMS and Investigation of Antibacterial Activity against Pathogenic Bacteria
Source: Molecules. 2023 May 7;28(9):3937. doi: 10.3390/molecules28093937 (PMC10180426; doi:10.3390/molecules28093937)
Supplement: Supplementary file 1 [file molecules-28-03937-s001.zip › molecules-2376973-supplementary.pdf]

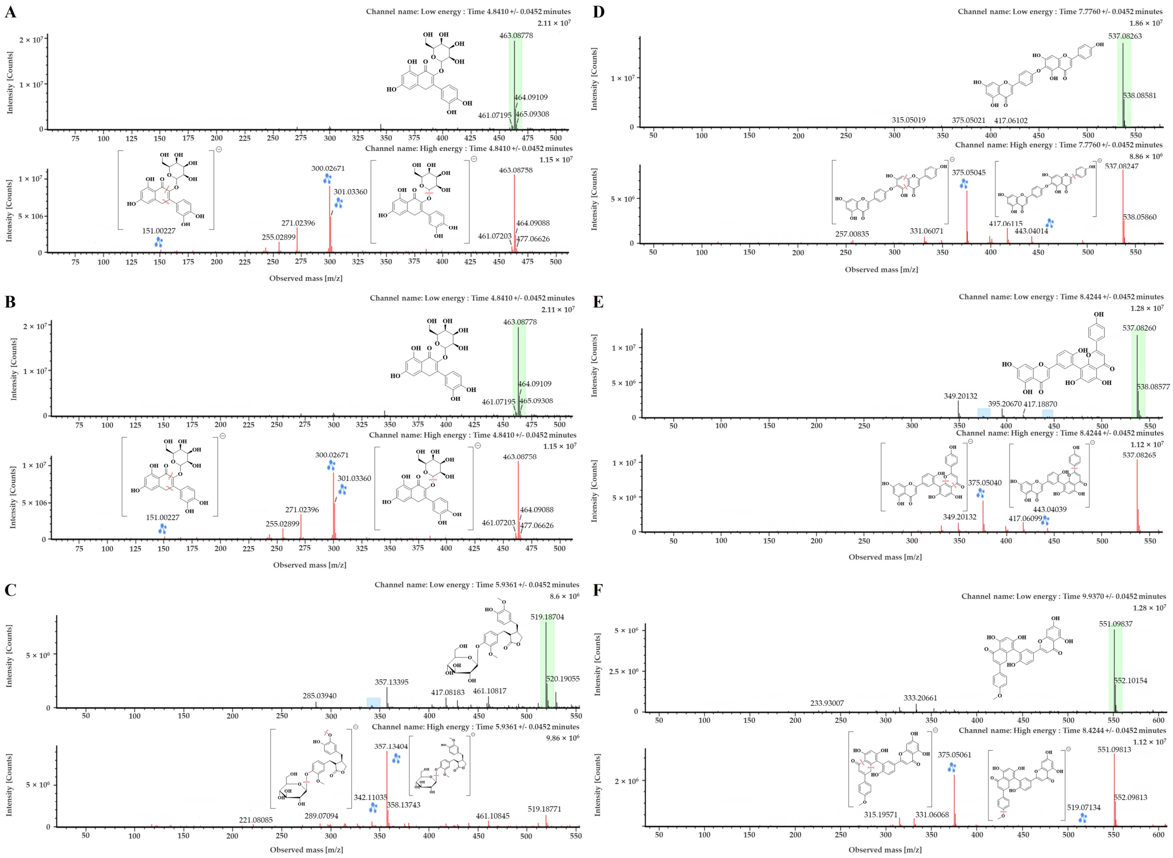

Figure S1: Fragment ion pattern isoquercetin (A), quercetin-3-*O*-α-L-rhamnoside (B), matairesinoside (C), hinokiflavone (D), amentoflavone (E), and podocarpusflavone A (F) using mass spectra obtained by UPLC-QTOF-MS.
